# Supplementary material for: Photolipid excitation triggers depolarizing optocapacitive currents and action potentials
Source: Nat Commun. 2024 Feb 7;15:1139. doi: 10.1038/s41467-024-45403-y (PMC10850502; doi:10.1038/s41467-024-45403-y)
Supplement: Supplementary file 1 — Supplementary Info [file 41467_2024_45403_MOESM1_ESM.pdf]

Supplementary to: Photolipid excitation triggers depolarizing optocapacitive currents and action potentials

Carlos A. Z. Bassetto Jr[1,#], Juergen Pfeffermann[2,#], Rohit Yadav[2,#], Simon Strassgschwandtner[2], Toma Glasnov[3], Francisco Bezanilla[1,4,*], Peter Pohl[2,*]

[1]Department of Biochemistry and Molecular Biology, The University of Chicago, Chicago, IL 60637, USA; [2]Institute of Biophysics, Johannes Kepler University Linz, Gruberstraße 40, 4020 Linz, Austria; [3]Karl-Franzens-University, Graz, Austria; [4]Centro Interdisciplinario de Neurociencia de Valparaíso, Facultad de Ciencias, Universidad de Valparaíso, Valparaíso, Chile

[#] These authors contributed equally to the paper

[*] Corresponding authors: Francisco Bezanilla, fbezanilla@uchicago.edu; Peter Pohl, e-mail: peter.pohl@jku.at

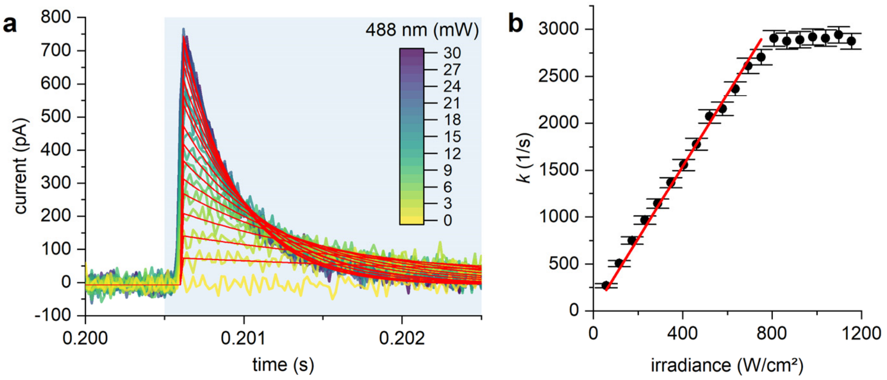

Supplementary Fig. 1: Power-dependence of I_{cap} decay rate. a, The power-dependent I_{cap} upon blue light exposure shown and described in Fig. 1e were fitted with a monoexponential decay model of the form: $y(t)=y_0$ for $t\leq0.2006$ s and $y(t)=A\times\exp(-(t-0.2006)\times k)+y_0$ for $t>0.2006$ s. A slight delay of ≈100 µs was introduced between the onset of light exposure and the start of exponential fitting (0.2006 s) to avoid the initial rise of the current which is not fully resolved due to analog filtering. **b,** The apparent rates of decay from the exponential fits in **a**, *k*, are plotted over irradiance estimated from power at the sample stage divided by area calculated from $1/e^2$-diameter of the blue laser profile. The data is fit between 60 and 750 Wcm^{-2} by a linear model of the form $y(x)=a\times x$, whereby $a=3.85$ $cm^2W^{-1}s^{-1}$ ($R^2>0.99$).

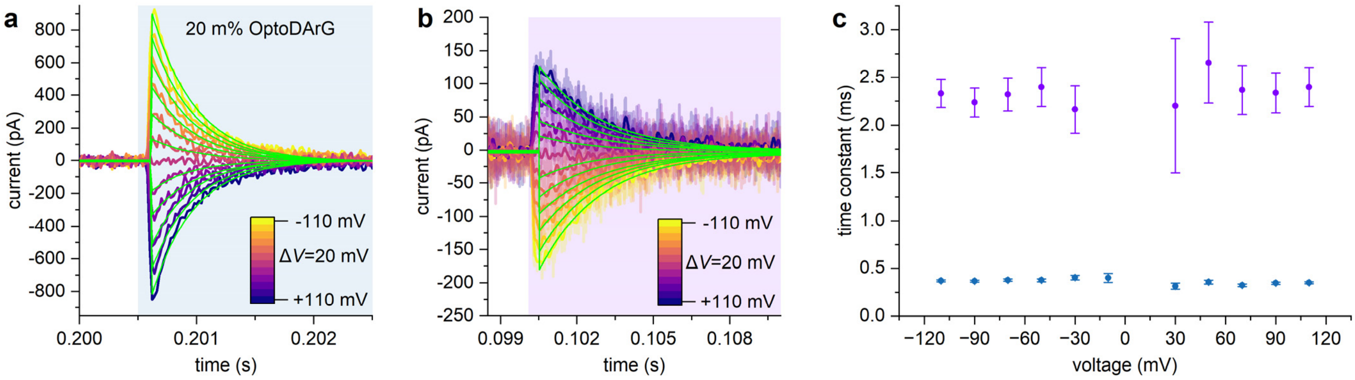

Supplementary Fig. 2: The rate constants of I_{cap} decay are voltage-independent, in accordance with the theoretical prediction. a,b, I_{cap} shown in Figs. 1c and 1d were fit as described in Supplementary Fig. 1a. For UV light-evoked I_{cap}, the exponential decay was fit starting from 0.1005 s. **c,** The resulting time constants, $1/k$, are plotted over applied voltage. Purple points correspond to UV light-evoked I_{cap} (**a**), blue points to blue light-evoked I_{cap} (**b**). Differences in the decay rates are due to differences in irradiance (UV light irradiance was lower than blue light irradiance) and differences in photoisomerization rate for the cis to trans and trans to cis transition (Arya, Jelken et al. 2020). As expected from Eq. 7, the rate of decay does not depend appreciably on the applied voltage.

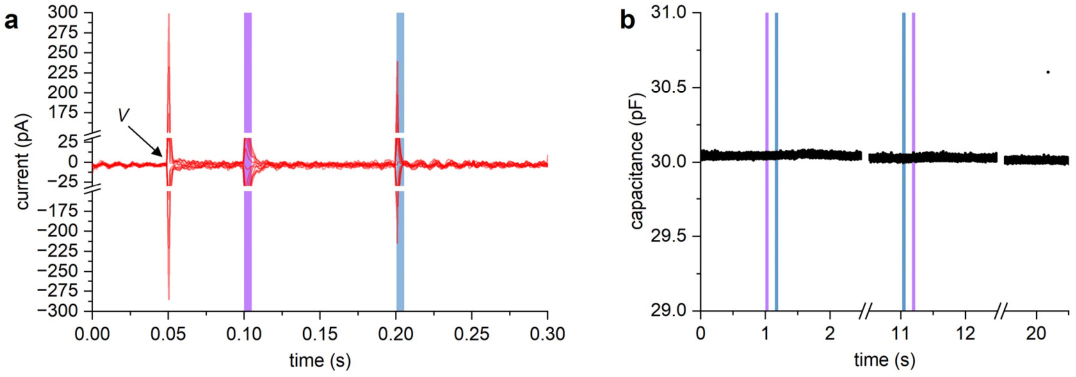

Supplementary Fig. 3: Controls. a, OptoDArG photoisomerization does not alter PLB conductance. The red traces are digitally filtered versions of the raw current traces from which Figs. 1c and 1d were prepared (the color coding according to power was omitted). As in Fig. 1, purple and blue background designate UV and blue light exposure, respectively. The arrow annotated *V* designates the application of voltage ranging from −110 mV to +110 mV with Δ*V*=20 mV, as described in the caption to Fig. 1; prior to 50 ms, *V* was 0 mV. Note that the voltage jump- and photoisomerization-evoked capacitive currents are truncated to allow for a more focused view of the baseline. **b,** In the absence of OptoDArG, capacitance does not change upon blue or UV light exposure. The recording was done as in Fig. 2a but on a PLB folded from 100% *E. coli* PLE.

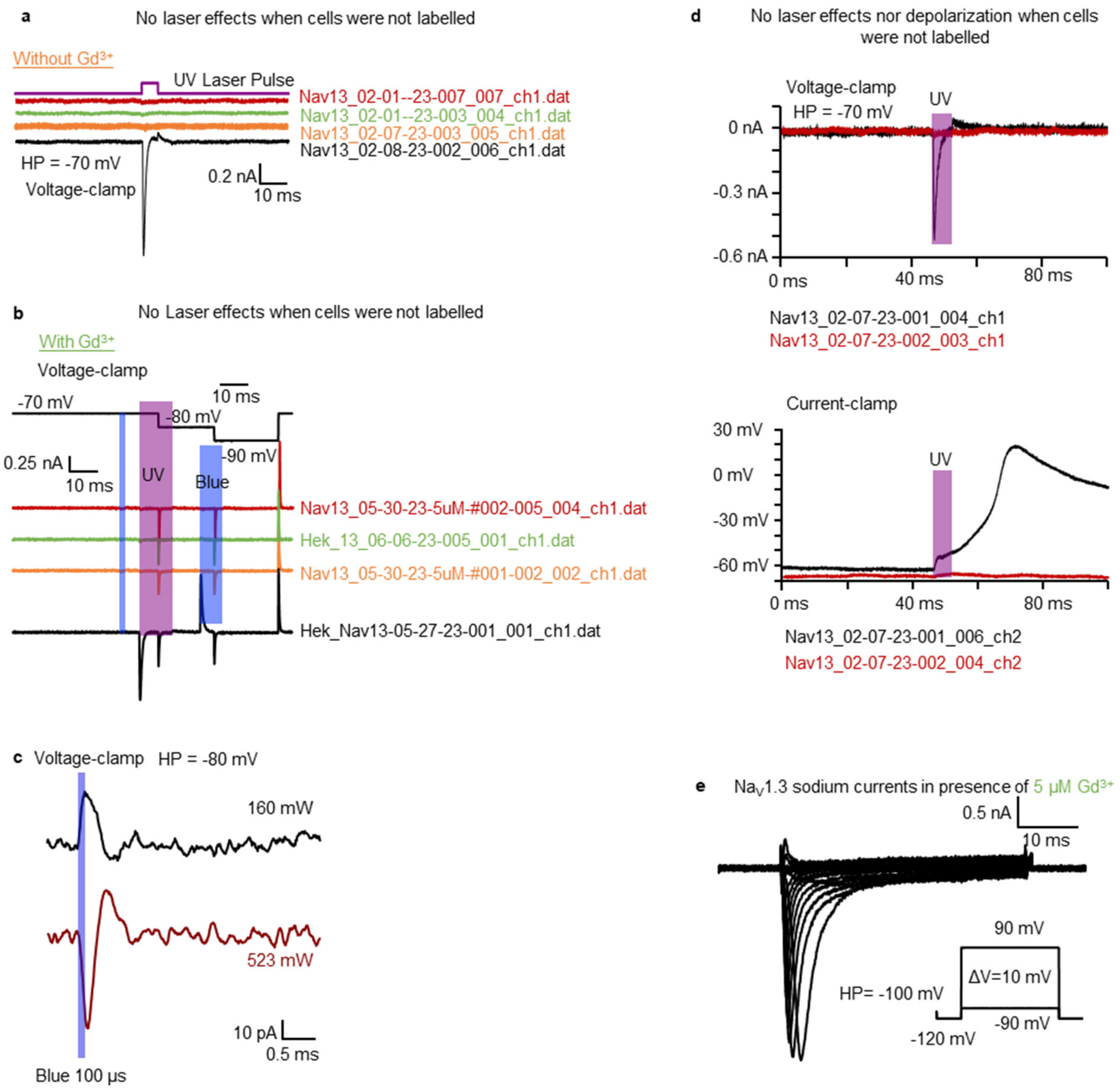

Supplementary Fig. 4: No optocapacitive current is present when cells were not labeled with OptoDArG and 5 μM Gd^{3+} does not affect Na^+ currents through $Na_V1.3$. a, Without OptoDArG labeling, no optocapacitive currents were induced by UV light exposure, indicating the absence of photothermal effects due to UV light itself. The red, green, and orange are representative current traces for cells that did not have OptoDArG, and the black for a cell that had OptoDArG for conditions in the absence (**a**) and in the presence of Gd^{3+} (**b**). In (**a**) the voltage was held at −70 mV and a single UV pulse was applied as indicated. In (**b**) the voltage protocol is inset at the top of the panel and the duration of exposure to laser light is shown by the rectangles. **c**, Thermal effects induced by blue laser when power exceeds 200 mW. The top trace (160 mW) shows light-induced capacitance changes by OptoDArG; the bottom trace (523 mW) displays the photothermal effect. Traces are from the same cell, and they were primed by UV prior to the indicated blue light exposure. **d,** No depolarization is observed when cells did not have OptoDArG incorporated. The red and black traces are recordings in voltage-clamp (top) and current-clamp (bottom) for cells in the absence and presence of OptoDArG, respectively. More details regarding the power used in our experiments can be found in Supplementary Table 1 and 2. The current and voltage-clamp experiments were from the same cell. **e,** Na^+ currents elicited by the voltage protocol inset in **e,** and in presence of 5 μM Gd^{3+}.

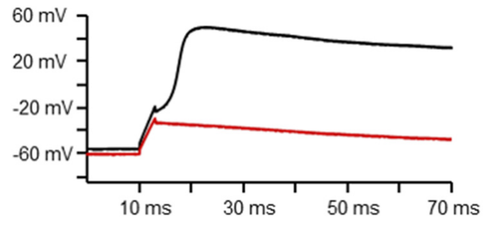

Supplementary Fig. 5: Cells expressing $Na_V1.3$ required large depolarization to elicit an AP. The black trace represents an AP elicited by injecting current and the red trace failure to fire even though the depolarizations induced were similar. Both depolarizations were induced by injecting current.

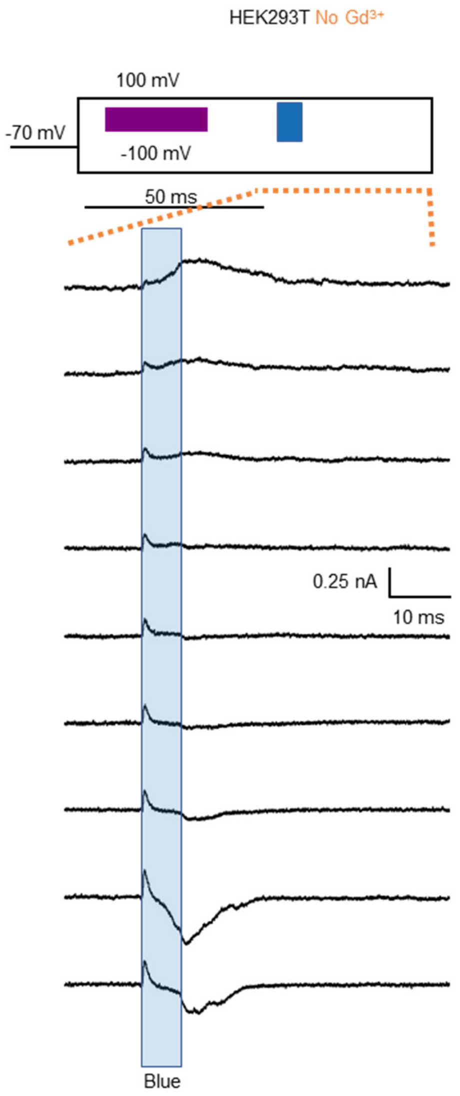

Supplementary Fig. 6: Mechanosensitive currents evoked upon switching to trans-OptoDArG in blank HEK293T cells. Currents were elicited using non-transfected (blank) HEK293T (ATCC - CRL-1573) cells at different voltage. A 30 ms UV laser pulse, indicated as a light blue square (30 mW), was applied before to prime the cis to trans transition induced by the blue laser (160 mW), indicated as blue square in the voltage protocol inset. The voltage protocol is illustrated in the inset and the time shown is indicated by orange dashed lines.

Table1: Success of AP generation in the absence of Gd^{3+} and labeling conditions.

Recording file	peak current by UV at −70 mV (nA)	laser power (mW)		Δ*V* (mV) by UV	AP by UV	labeling	Δ*V* (mV) by injecting current
		UV	Blue				
Nav13_02-01--23-001_006_ch1.dat	-0.218201	100	152	10.68	No	Yes	not measured
Nav13_02-01--23-003_004_ch1.dat	-0.0427246	100	152	not tried	not tried	No	
Nav13_02-01--23-005_003_ch1.dat	-0.108337	100	152	7.02	Yes	Yes	not measured
Nav13_02-01--23-006_002_ch1.dat	-0.0869751	100	152	7.69	Yes	Yes	not measured
Nav13_02-01--23-007_007_ch1.dat	-0.0442505	100	152	not tried	not tried	No	
Nav13_02-02-23-001_005_ch1.dat	-1.10321	80	152	16.78	Yes	Yes	not measured
Nav13_02-02-23-002_005_ch1.dat	-0.637817	80	152	14.95	No	Yes	not measured
Nav13_02-02-23-003_004_ch1.dat	-0.552368	80	152	14.04	No	Yes	not measured
Nav13_02-07-23-001_005_ch1.dat	-0.276184	100	152	7.93	No	Yes	not measured
Nav13_02-07-23-002_003_ch1.dat	-0.0518799	100	152	no depolarization	No	No	44.25
Nav13_02-07-23-003_005_ch1.dat	-0.0473022	100	152	not tried	not tried	No	
Nav13_02-07-23-004_003_ch1.dat	-0.418091	100	152	7.32	No	Yes	not enough sodium current
Nav13_02-07-23-006_003_ch1.dat	-0.131226	100	152	not tried	not tried	Yes	
Nav13_02-08-23-002_006_ch1.dat	-0.917053	100	152	10.07	Yes	Yes	21.97
Nav13_02-08-23-001_003_ch1.dat	-0.0198364	100	152	not tried	not tried	No	
HEK_Nav13_05-23-23-003_002_ch1.dat	-0.561523	98	60	11.14	No	Yes	not measured
HEK_Nav13_05-25-23-#2-005_003_ch1.dat	-0.141907	124	60	not tried	not tried	little	
HEK_Nav13_05-25-23-#2-007_002_ch1.dat	-0.0762939	124	60	not tried	not tried	little	
HEK_Nav13_05-25-23-#2-008_002_ch1.dat	-0.177002	124	60	not tried	not tried	little	
HEK_Nav13_05-25-23-#3-010_003_ch1.dat	-0.288391	124	60	8.39	No	Yes	31.01
HEK_Nav13_05-25-23-002_006_ch1.dat	-0.617981	98	60	15.01	No	Yes	not measured
HEK_Nav13_05-25-23-003_004_ch1.dat	-0.128174	124	152	not tried	not tried	little	

Table 2: Success of AP generation in the presence of Gd^{3+} and labeling conditions.

Recording file	Gd^{3+} (μM)	peak current by UV at −70 mV (nA)	laser power (mW)		Δ*V* (mV) by UV	AP by UV	labeling	Δ*V* (mV) by Injecting current
			UV	Blue				
Hek_Nav13-05-27-23-#002_10uMGd-013_005_ch1.dat	10	-0.695801	120	60	15	No	Yes	30mV
Hek_Nav13-05-27-23-001_001_ch1.dat	50	-1.9455	124	150	17.73	No	Yes	not measured
Hek_Nav13-05-27-23-003_003_ch1.dat	50	-0.58136	98	12	17	No	Yes	not measured
Hek_Nav13-05-27-23-#002-20uM-011_006_ch1.dat	20	-1.04218	98	60	18.98	No	Yes	36.87
Hek_Nav13-05-27-23-20uM-008_002_ch1.dat	20	-0.398254	124	60	8.18	No	Yes	not measured
Hek_Nav13-05-27-23-#002-20uM-009_004_ch1.dat	20	-0.198364	124	60	6.81	No	Yes	not measured
Nav13_05-30-23-5uM-#001-002_002_ch1.dat	5	-0.0137329	124	150	not tried	not tried	No	
Nav13_05-30-23-5uM-#001-003_003_ch1.dat	5	-0.294495	124	150	not tried	not tried	Little	
Nav13_05-30-23-5uM-#001-004_005_ch1.dat	5	-0.187683	124	150	not tried	not tried	No	
Nav13_05-30-23-5uM-#002-005_004_ch1.dat	5	-0.0167847	124	150	not tried	not tried	No	
Nav13_05-30-23-5uM-#002-006_003_ch1.dat	5	-0.0534058	124	150	not tried	not tried	No	
Nav13_05-30-23-5uM-#002-009_004_ch1.dat	5	-0.0534058	124	150	not tried	not tried	little	
Nav13_05-30-23-5uM-#003-010_002_ch1.dat	5	-0.125122	98	12	not tried	not tried	little	
Nav13_05-30-23-5uM-#003-012_005_ch1.dat	5	-0.0778198	120	60	not tried	not tried	little	
Nav13_05-30-23-5uM-#003-011_005_ch1.dat	5	-0.439453	120	60	6.21	No	Yes	not measured
Nav13_05-30-23-5uM-#003-013_005_ch1.dat	5	-0.265503	124	150	5.83	No	Yes	35.13
Nav13_05-30-23-5uM-#003-014_002_ch1.dat	5	-0.163269	124	150	3.78	No	Yes	not measured
Nav13_05-30-23-5uM-#003-015_002_ch1.dat	5	-0.0732422	124	150	not tried	not tried	little	32.14
Nav13_05-30-23-5uM-#003-016_004_ch1.dat	5	-0.624084	124	150	12.56	No	Yes	not measured
Nav13_05-30-23-5uM-017_003_ch2.dat	5	not recorded	124	150	8.45	Yes	Yes	not measured
Hek_13_06-06-23-001_004_ch1.dat	10	-0.135803	124	150	not tried	not tried	little	
Hek_13_06-06-23-007_002_ch1.dat	10	-0.349426	124	150	6.4	No	Yes	39.25
Hek_13_06-06-23-008_002_ch1.dat	10	-0.500488	124	150	9.22	No	Yes	not measured
Hek_13_06-06-23-003_002_ch1.dat	10	-0.219727	124	150	not tried	not tried	No	
Hek_13_06-06-23-005_001_ch1.dat	10	-0.0198364	124	150	not tried	not tried	No	
Hek_13_06-07-23-#Dish_2-010uM_GdCl-005_003_ch1.dat	10	-0.300598	124	150	not tried	not tried	little	
Hek_13_06-07-23-001_005_ch1.dat	10	-0.489807	124	12	3.78	No	Yes	23.41

Hek_13_06-07-23-002_002_ch1.dat	10	-3.66211	124	150	not tried	not tried	Yes	
Hek_13_06-07-23-003_009_ch1.dat	10	-0.668335	124	150	9.19	Yes	Yes	not measured
Hek_13_06-07-23-0015uM_GdCl_001_ch1.dat	15	-1.4267	124	150	not tried	not tried	Yes	
